# Characterization and Antimicrobial Resistance of Bacteria Causing Subclinical Mastitis in Dairy Cows in the Upper Cheliff Region, Northern Algeria

**DOI:** 10.3390/antibiotics14121190

**Published:** 2025-11-22

**Authors:** Ahmed Khelili, Rachid Achek, Mohammed R. Abdullah, Abdelkadir Karim, Ibrahim Nabi, Amira A. Moawad, El-Hassen Lankri, Evgeny A. Idelevich, Karsten Becker

**Affiliations:** 1 Laboratory of Bioresources Natural Local, Department of Agronomy, Faculty of Nature and Life Sciences, University of Hassiba Ben-Bouali, Chlef 2010, Algeria; a.khelili@univ-chlef.dz (A.K.); e.lankri@univ-chlef.dz (E.-H.L.); 2 Faculty of Nature and Life and Earth Sciences, Djilali-Bounaama University, Khemis-Miliana 44001, Algeria; a.karim@univ-dbkm.dz; 3 Laboratory of Food Hygiene and Quality Assurance System, Higher National Veterinary School, Algiers 16000, Algeria; 4 Friedrich Loeffler-Institute of Medical Microbiology, University Medicine Greifswald, 17475 Greifswald, Germany; mohammed.abdullah@med.uni-greifswald.de (M.R.A.); evgeny.idelevich@med.uni-greifswald.de (E.A.I.); karsten.becker@med.uni-greifswald.de (K.B.); 5 Laboratory of Biotechnology and Valorisation of Biological Resources BVRB, Faculty of Sciences, Yahia Farès University, Médea 26000, Algeria; nabi.ibrahim@univ-medea.dz; 6 Institute of Bacterial Infections and Zoonoses, Friedrich-Loeffler-Institute, 07743 Jena, Germany; amira.moawad@fli.de; 7 Animal Health Research Institute (AHRI), Agriculture Research Center (ARC), Giza 12618, Egypt; 8 Institute of Medical Microbiology, University Hospital Münster, 48149 Münster, Germany

**Keywords:** subclinical mastitis, dairy cows, antimicrobial resistance, milk production, reproductive performance, Upper Cheliff

## Abstract

**Background/Objectives:** Subclinical mastitis is a common and economically significant infection in dairy cows. This study aimed to assess the prevalence and antimicrobial susceptibility of bacteria causing subclinical mastitis in dairy cows in the Upper Cheliff Region, Northern Algeria, and to investigate the effects of subclinical mastitis on milk production and reproductive performance. **Methods:** A total of 263 cows from 23 farms were screened for subclinical mastitis using the California Mastitis Test (CMT) and sampled for isolation and identification of bacteria by MALDI-TOF MS. Antimicrobial susceptibility testing (AST) was performed using the Vitek 2 system and disk diffusion method. Methicillin resistance in staphylococci and mammaliicocci was confirmed by *mec*A/*mec*C detection. **Results:** The results revealed a prevalence of subclinical mastitis of 58.9% at the cow-level and 31.1% at the quarter-level. The most prevalent microorganisms identified were *Enterococcus faecium* (*E. faecium*) (24.4%) and *Enterococcus faecalis* (*E. faecalis*) (20.5%), followed by non-*aureus* staphylococci (NAS) (16.9%), *Escherichia coli* (*E. coli*) (7.9%) and *Staphylococcus aureus* (*S. aureus*) (7.08%). Risk factors significantly associated with the prevalence of subclinical mastitis included parity, lower milk production (<12 L/day), poor animal cleanliness and a history of previous mastitis. Reproductive performance was significantly impaired in cows with subclinical mastitis, cows with longer calving-to-first-service intervals (130 vs. 102.7 days; *p* < 0.0001), more services per conception (2.5 vs. 1.9; *p* < 0.0001) and a lower pregnancy rate at first service (24.5% vs. 48.1%; *p* < 0.0001). Furthermore, 43.2% of cows with subclinical mastitis required three or more inseminations to achieve pregnancy. AST showed a low resistance rate for the antimicrobial agents most commonly used in human and veterinary medicine. Coagulase-negative staphylococci (CoNS) showed remarkable rates of methicillin-resistance (22.2%), as well as resistance towards fosfomycin (37.8%) and tetracycline (31.1%). A substantial proportion of *E. coli* isolates exhibited resistance to piperacillin (40%) and ciprofloxacin (15%). All *S. aureus* isolates were classified as MSSA without detection of *mec*A and *mec*C genes. **Conclusions:** The results of this study demonstrated that subclinical mastitis is prevalent in the Upper Cheliff region and is associated with reduced milk production and reproductive performance. The detection of pathogenic and resistant microorganisms in milk is alarming and requires effective management strategies to control subclinical mastitis and improve dairy farm productivity.

## 1. Introduction

Bovine mastitis is an inflammatory disease that affects the mammary gland tissue, primarily caused by intramammary bacterial infections. The most common pathogens causing mastitis are classified into contagious mastitis due to spread from other infected quarters (e.g., *Streptococcus agalactiae*, *Streptococcus dysgalactiae*, *S. aureus,* CoNS, *Corynebacterium bovis* and *Mycoplasma bovis*) and environmental mastitis caused by organisms from the surrounding environment (e.g., *Enterobacterales*, *Pseudomonas aeruginosa*, enterococci, environmental streptococci and fungi) [1,2]. Mastitis can be classified into two categories based on the degree and severity of inflammation: clinical mastitis and subclinical mastitis [3]. Clinical mastitis is easily recognized by sudden onset and visible abnormalities, such as the presence of blood in the milk, swelling and redness of the udder, and fever in the cow. In contrast, subclinical mastitis is an asymptomatic infection without visible changes to the udder and is thus more difficult to detect. However, subclinical mastitis results in reduced milk production with corresponding production losses, although not to the same extent as in clinical mastitis. Furthermore, subclinical mastitis leads to changes in milk parameters as well as to an increase in the number of somatic cells in the milk [4]. Incidence and prevalence of mastitis vary among countries and herds, in which clinical mastitis represents only a small fraction, subclinical mastitis being far more prevalent [5,6,7].

Differences in the occurrence of subclinical mastitis and the distribution of mastitis-causing pathogens in the herds were associated with various risk factors. Several studies have thoroughly investigated the risk factors for subclinical mastitis. The key cow-specific risk factors included age, parity, lactation phase, genetics and breed, milk production, immune system and body condition score [6,8,9]. However, factors related to herd management were hygiene conditions, milking technique, production type and nutrition [10,11]. Other environmental factors such as climate and season are also considered [12,13].

Subclinical mastitis is one of the leading causes of economic losses in dairy herds worldwide [14]. It affects cow welfare and causes significant financial losses through reducing milk production, premature culling, veterinary treatments and medication, milk discarded due to antibiotic residues, and imposing penalties on milk quality based on elevated somatic cell counts (SCC) or bacterial load [11,15,16,17]. It not only affects milk production but also disrupts the systemic immune response and induces endocrine changes, all of which can negatively influence reproductive performance [18]. Cows with mastitis experience delayed estrus, lower pregnancy rates, impaired embryonic development, and an increased risk of abortion [19]. Additionally, mastitis can affect reproduction by damaging follicles, hindering oocyte growth or function, and reducing the cow’s ability to ovulate. Consequently, it increases the following reproductive parameters: calving-to-first-service interval, days open, number of services per conception, and number of artificial inseminations per conception [20].

The primary strategy for curing mastitis in dairy cattle involves the use of antibiotic treatment, with β-lactams (particularly penicillins, streptomycin and cephalosporins), tetracyclines and macrolides (erythromycin) being the most commonly administered [21,22]. However, the widespread and abusive use of these antimicrobial agents in veterinary medicine has contributed to the development of antimicrobial resistance (AMR) in various pathogenic bacteria [23]. The unnecessary use of broad-spectrum antibiotics without accurately identifying the causative agent or conducting prior antimicrobial susceptibility testing can accelerate the emergence of resistance. Resistant bacteria may spread within populations either vertically through clonal transmission or horizontally via gene transfer [24]. As a result, antimicrobial resistance not only reduces the effectiveness of treatment in animals but also poses a significant public health risk, such as methicillin-resistant (MR) strains of *S. aureus*, particularly livestock-associated methicillin-resistant *S. aureus* (LA-MRSA) [25,26,27]. With regard to cattle farming, this risk arises from the potential transmission of resistant bacteria to humans through the consumption of unpasteurized milk from infected cows or even milk containing antibiotic residues [21,28].

In Algeria, the dairy cattle herds are primarily concentrated (60%) in the coastal fringe and northern interior plains, specifically in the Setif, Metidja, and Cheliff plains [29]. The Upper Cheliff plain is a key dairy-producing region, covering 28.000 hectares of irrigated agricultural land [30,31]. Dairy farms in this area are divided into large pilot farms with high head capacities and smaller private farms [32].

The success of self-sufficiency in dairy production within Algeria, particularly in this region, is hindered by various challenges that impact both efficiency and productivity. Key factors limiting dairy farm profitability and cow productivity include inadequate feeding and management practices, as well as issues related to disease and health management [33,34,35]. Subclinical mastitis stands out as a significant production-related disease in dairy cows, posing a substantial barrier to achieving optimal productivity [36,37,38]. The prevalence of this disease remains unknown and is likely underestimated due to the absence of a comprehensive national surveillance program encompassing diagnostics, treatment, and preventive measures. Consequently, both clinical mastitis and subclinical mastitis cases are often treated empirically, frequently without appropriate milk sampling for bacteriological analysis. This deficiency in pathogen-specific research impedes effective mastitis control and treatment strategies. Moreover, limited studies have examined the risk factors associated with subclinical mastitis in Algerian dairy herds and assessed its impact on dairy production and reproductive parameters. The aims of this study were to assess the prevalence and risk factors of subclinical mastitis in dairy cows in Algeria’s Upper Cheliff region, identify the main pathogens involved, investigate their antimicrobial susceptibility and evaluate the impact of subclinical mastitis on milk yield and reproductive status of a population of dairy cows.

## 2. Results

### 2.1. Prevalence of Subclinical Mastitis at Cows and Quarters

In this study, 263 cows raised in 23 farms were screened for subclinical mastitis using the CMT. The cow-level prevalence of subclinical mastitis was 58.9% (155 positive cows out of 263 tested), while the quarter-level prevalence was 31.1% (317 positive quarters out of 1020 functional quarters examined) and 3% of quarters were deemed blind and non-functional.

Among the total cows infected with subclinical mastitis, the distribution of infected quarters varied. A majority of the cows had one (35.5%) or two (34.2%) infected quarters (*p* < 0.0001), while a smaller proportion had more widespread infection across multiple quarters (Table 1).

The distribution of CMT scores in infected quarters showed that a significant proportion (54.9%) of the positive quarters had a CMT score of 3 (*p* < 0.0001), indicating a higher severity of mastitis. In contrast, 9.8% of the quarters had a CMT score of 1, suggesting a less severe infection. Only 2.2% of the infected quarters had a CMT score of 4, representing the most severe stage of subclinical mastitis, which generally tends towards clinical mastitis (Table 1).

The distribution of infected quarters by position in cows indicated that the highest rate of infection was found in the rear right quarter (29%), followed closely by the rear left quarter (25.9%). The front right and front left quarters had infection rates of 24.3% and 20.8%, respectively (Table 1).

### 2.2. Prevalence of Bacterial Species Found in Subclinical Mastitis

Microbiological analysis showed that a single bacterial species was detected in 71.6% (227/317) of all CMT-positive milk samples exhibiting positive bacterial culture, whereas 22.7% were culture-negative and 5.7% were deemed contaminated given more than two types of two different colony morphologies (Table 2).

The distribution of isolated species showed a high diversity. *E. faecium* and *E. faecalis* were the most prevalent (24.4% and 20.5%), respectively, followed by NAS (17.7%). *E. coli* and *S. aureus* were isolated from 20 (7.9%) and 18 (7.08%) samples, respectively. *Mammaliicoccus sciuri* was detected only in one sample. Species belonging to the genera *Streptococcus*, *Corynebacterium*, *Aerococcus* and *Lactococcus* were identified at low rates (Table 2). Regarding the relationship between bacterial isolation and CMT scores, over half (54.3%) of the isolates were obtained from milk samples with a score of 3, indicating a clear infection of the mammary parenchyma. This trend was particularly evident for *E. coli* and *S. aureus*, with 100% and 63.1% of isolates, respectively, coming from samples with a score of 3 in CMT.

### 2.3. Antimicrobial Susceptibility

The results of susceptibility testing demonstrate generally low resistance levels among the tested bacterial isolates. All *S. aureus* isolates were methicillin-susceptible and showed complete susceptibility (100%) to tested antibiotics, including benzylpenicillin, oxacillin, vancomycin and linezolid (Table 3). None of the *S. aureus* isolates were found to contain the *mec*A and *mec*C genes. In contrast, CoNS showed remarkable rates of methicillin resistance (22.2%). Resistance to other antimicrobial classes in CoNS was found particularly in MR isolates, including resistance to fosfomycin (total, 37.8%; MR-CoNS, 50%), tetracycline (total, 31.1%; MR-CoNS, 70%), erythromycin (total, 15.5%; MR-CoNS, 60%) and clindamycin (total, 13.3%; MR-CoNS, 40%). Enterococci isolates showed no resistance to key antibiotics such as vancomycin and teicoplanin, with only very low resistance to levofloxacin and linezolid (0.8%). *E. coli* was completely susceptible (100%) to carbapenems and third-generation cephalosporins but showed resistance to piperacillin (40%), penicillin (35%) and ciprofloxacin (15%). Moreover, streptococci had high susceptibility across all tested antibiotics, including vancomycin, linezolid, and cefotaxime.

### 2.4. Risk Factors Associated with Subclinical Mastitis

Parity was identified as a significant risk factor associated with subclinical mastitis; multiparous cows exhibited a higher positivity rate (63.2%) for mastitis compared to primiparous cows (41.2%), with a significant odds ratio (OR = 0.4; *p* < 0.004). The highest mastitis rate was observed in Holstein cows (68.2%) compared to the other breeds: Montbéliarde (59.5%), Fleckvieh (46.2%), and crossbreed cows (48.5%). However, the statistical analysis showed no significant effect of breed as a risk factor for mastitis (*p* = 0.267). The farming system and milking method also did not show significant associations with subclinical mastitis (*p* = 0.067 and *p* = 0.131, respectively), though cows kept in free-stall systems had the highest positivity rate of subclinical mastitis compared with those raised in an intensive and/or semi-intensive system (Table 4).

Significant findings were observed regarding daily milk production, where there was a higher positivity rate (91.3%) of subclinical mastitis among cows producing less than 12 L of milk, compared to those producing more than 12 L (37.7%; *p* < 0.0001). Animal cleanliness score was strongly correlated with mastitis occurrence (*p* < 0.0001); cows with higher scores (dirty, score 3) had a 100% positivity rate, compared to 46 (33.3%) in those with cleaner conditions (score 1). The milking method and the application of disinfection before and/or after milking showed no significant effect on mastitis (*p* > 0.05); despite that, cows mechanically milked with the application of disinfection were more likely to develop subclinical mastitis. The lactation stage was significantly associated with the prevalence of subclinical mastitis (*p* < 0.0001); cows in the mid and late stages of lactation had a higher prevalence of subclinical mastitis than cows in the early-lactation stage. The previous history of mastitis significantly (*p* < 0.0001) impacted the development of subclinical mastitis, with 95.6% of cows having a precedent of mastitis developing subclinical mastitis in contrast to 51.4% in those without a previous history of mastitis.

### 2.5. Effect of Subclinical Mastitis on Dairy Production and Reproductive Performances

Cows with subclinical mastitis had a longer calving-to-first-service interval (CFSI) (130 ± 39.9 days) compared to those without subclinical mastitis (102.7 ± 23.3 days) (*p* < 0.0001). The number of services per conception (NSPC) was significantly higher in subclinical mastitis-positive cows (2.5 ± 1.4) compared to subclinical mastitis-negative cows (1.9 ± 1) (*p* < 0.0001). The calving-to-conception interval (CCI) was notably shorter in subclinical mastitis-negative cows (124.4 ± 28.3 days) than in cows with subclinical mastitis (164.7 ± 67.6 days) (*p* < 0.0001). Then, the pregnancy rate at first service or first artificial insemination (PRFS) was significantly lower in subclinical mastitis-positive cows (24.5%) compared to those negative for subclinical mastitis (48.1%) (*p* < 0.0001). In addition, 43.2% of cows with subclinical mastitis required ≥3 inseminations to have gestation compared to those without subclinical mastitis (Table 5).

Regarding the impact of the number of infected quarters on the reproductive performance in cows having subclinical mastitis, the results showed that fertility parameters deteriorate significantly (*p* < 0.0001) with the number of positive quarters, in which cows that had four infected quarters had longer CFSI and required 4.4 ± 1.9 inseminations to become pregnant (Table 6).

## 3. Discussion

Subclinical mastitis remains one of the most prevalent diseases in dairy farming, which leads to significant financial losses [14]. It impacts the cow’s health and reduces milk yield and quality. In the absence of systematic diagnostic testing, subclinical mastitis pathogens can contaminate dairy processing and may even pose a risk of foodborne illness to consumers [2,39].

The present study addressed an assessment of the prevalence of and identified bacterial species causing subclinical mastitis in dairy cows in the Upper Cheliff plain region, Northern Algeria, as well as the impact on dairy production and reproductive performances.

The present findings showed that more than half (58.9%) of dairy cows tested were suffering from subclinical mastitis. Similar results were obtained previously in Algeria [37], Tunisia [40] and Rwanda [41], which reported prevalence rates of 62.8%, 60.3% and 60%, respectively. However, other studies carried out in Algeria [36,38,42,43] reported lower prevalence of subclinical mastitis (40%, 37.7%, 45.9% and 34.9%, respectively). In Egypt, they also reported a lower prevalence (46%) of subclinical mastitis [44]. Regarding prevalence at the quarter level, our results showed 31.1% were in agreement with previous studies conducted in Algeria, in which prevalence was estimated as 28.8% [45], 27.2% [38], 31.1% [37] and 24.5% [43].

The distribution of bacterial species involved in subclinical mastitis varies across herds, regions, and countries and is apparently influenced by management practices and environmental factors that include hygiene conditions of cows and farms, CMT scores, and the methods used for isolation and identification. It is well known that skin-associated Gram-positive bacteria such as staphylococci and streptococci are primarily responsible for subclinical mastitis [46,47,48]. Additionally, environmental bacteria such as enterococci and *Enterobacterales* have also been implicated [49].

In the present study, enterococci (*E. faecalis* and *E. faecium*) were the most frequently isolated, followed by NAS and *S. aureus*, in concordance with Cheng and Han [12]. Previous investigations reported the isolation of *S. aureus* in 29.8% [43] and 35.6% [36] of subclinical mastitis cases in Algeria and 44.9% in Egypt [44]. The detection of staphylococci might reflect the poor hygiene management, in which these bacteria are transmitted from cow to cow through contaminated milkers’ hands or via contaminated milking machines.

The role of various pathogens in subclinical or clinical mastitis is influenced by the cow’s immune system and the virulence factors of the isolates. In the present study, the CMT provides an indication of the SCC and reveals the degree of infection. In fact, the distribution of pathogens in subclinical mastitis varies according to the SCC as evaluated by the CMT [50]. In this study, we observed a relationship between the increase in CMT score and the presence of *E. coli* and *S. aureus*; 95% of *E. coli* and 61.1% of *S. aureus* were isolated from milk samples scored 3 on the CMT. Since the clinical mastitis is primarily caused by contagious (*S. aureus* and *S. agalactiae*) and environmental (*E. coli*) pathogens, Kaczorowski et al. [47] suggest that healthy cows with high subclinical mastitis can serve as a reservoir for mastitis pathogens and may subsequently develop into clinical mastitis in the absence of appropriate treatment.

CoNS are very prevalent in bovine intramammary infection, especially in dairy cows [51]. In the present study, 45 (17.7%) isolates belong to recent or former (i.e., species of the recently established *Mammaliicoccus* genus). CoNS were identified, mostly belonging to the species *S. epidermidis*, *S. simulans*, *S. haemolyticus* and *S. chromogenes*. The diversity and distribution of CoNS and NAS-positive staphylococci varied from country to country, as reported by De Buck et al. [52], with *S. chromogenes* is the most prevalent species causing subclinical mastitis, even with low or high SCC levels, followed by *S. simulans*, *S. xylosus*, *S. haemolyticus*, and *S. epidermidis*. In Egypt, CoNS were isolated at a rate of 37.1% and *S. xylosus* was the most prevalent species (*n* = 64; 35.4%) followed by *S. chromogenes* and *S. Epidermidis* [44]. In a retrospective study conducted in Germany between 2014 and 2023, CoNS were the most common species isolated from the culture-positive samples (30%), followed by *S. aureus* (19%) [53]. In Algerian studies, the prevalence of CoNS was reported at the level of the group without differentiation of species. We noted that our study was the first to use MALDI-TOF MS as an identification method and screen all the bacterial species found in milk samples of subclinical mastitis.

The present findings showed that 72 (22.7%) positive CMT samples were negative for bacterial culture. Other studies [38,41,44] found that 17.2%, 3.3% and 9.6% of CMT-positive samples did not yield positive cultures, respectively. The interpreting of CMT-positive/negative culture must consider various factors, as suggested by Dingwell et al. [54]. Primarily, the sensitivity (82.4%) and specificity (80.6%) of a positive CMT are highest on the fourth day of lactation; outside this period, both sensitivity and specificity can be lower due to the subjective nature of CMT interpretation. In addition, George et al. [55] reported that CMT scores tend to be higher in the first and last periods of lactation. Hence, other conditions, such as traumatic reticulo-peritonitis, may also release immune cells into the milk, resulting in a positive CMT in cows with healthy udders [55]. On the other hand, certain mastitis-causing pathogens, such as species of *Listeria*, *Mycoplasma* and fungi, may not grow on several standard culture media, as they require specific conditions for growth [44].

The prevention and control of mastitis in dairy cows require the implementation of various strategies, among which the use of antimicrobial agents remains an effective therapeutic approach. In Algerian farms, antimicrobial agents are often applied routinely to treat clinical mastitis without prior antimicrobial susceptibility testing. Previous studies conducted in Algeria have reported higher antimicrobial resistance rates in staphylococci species. In eastern Algeria, Zaatout et al. [42] found that all *S. aureus* isolates from dairy cattle were multidrug-resistant, showing particularly high resistance to penicillin, tulathromycin, and nalidixic acid. In western Algeria, Bouzidi et al. [56] reported that 19/62 (30.64%) of isolates collected from subclinical bovine mastitis cases were multidrug-resistant, in which 59 out of 62 were resistant to penicillin. Similarly, in the north-central region of Algeria, Akkou et al. [43] reported that 88.05% of *S. aureus* isolates from clinical mastitis cases were resistant to at least one of the tested antibiotics, with particularly high resistance rates to penicillin (86.5%) and tetracycline (14.9%). Regarding *E. coli* isolates, our findings align with those of Tahar et al. [57], who reported resistance rates of 13.5% to both penicillin and ciprofloxacin in *E. coli* isolated from clinical mastitis cases in Algeria. Additionally, a recent study by Khasapane et al. [23] in South Africa reported a significantly higher resistance to ciprofloxacin in *E. coli* isolates (70%), even though it was higher than our prevalence.

In the current study, most bacterial isolates were found to be susceptible to a range of antibiotic agents. However, moderate resistance was observed in CoNS (fosfomycin, oxacillin, tetracycline) and *E. coli*, particularly to piperacillin and ciprofloxacin. This study is the first to investigate subclinical mastitis in dairy cows in the Upper Cheliff region of northern Algeria. We observed that screening for subclinical mastitis was not reported as part of the routine husbandry and cattle breeding management programs in all the farms included in the present study. In contrast, a limited number of clinical mastitis cases were identified based on visible symptoms and were treated with the following antibiotics: penicillin-streptomycin, cefalexine, cefacetrile, oxytetracycline, erythromycin, kanamycin, rifaximin, tilmicosin, and cefalexine-kanamycin. Additionally, we found that uncured cows were removed from herds through sale or uncontrolled slaughter. These findings highlight the need for further comprehensive surveys to accurately assess the true prevalence of mastitis in cattle herds and to identify the risk factors contributing to antimicrobial resistance. Furthermore, significant instability in herd composition has been reported in the Upper Cheliff region. According to Djermoun et al. [58], approximately 70% of dairy herds in the area were partially or entirely renewed between 2010 and 2017.

In addition, during our survey of bovine farms, we examined the distribution of farms based on their legal status: pilot modern farms and private farms. We found that most cases of subclinical mastitis were reported on pilot modern farms. These farms were characterized by better technical and logistical organization, stronger veterinary oversight, and integration into industrial dairy production circuits. According to Belkheir et al. [59], such farms benefit from streamlined management, modern breeding practices and regular access to veterinary services. As a result, no antibiotic treatment was administered during the lactation or dry periods to prevent subclinical mastitis cases.

In contrast, previous studies that reported high levels of antimicrobial resistance were conducted on individual cows from private farms. It is important to note that private farms typically follow more traditional breeding practices and are known for the habitual self-administration of antimicrobial agents for treatment or disease prevention—practices less commonly observed in pilot farms [60,61].

The results of the current study showed several risk factors associated with subclinical mastitis in cows. Overall, parity, daily milk production, and cleanliness emerged as major risk factors for subclinical mastitis. The incidence of subclinical mastitis was higher in multiparous cows than in primiparous cows, in concordance with [4,6,62]. The increase in subclinical mastitis prevalence with age and parity may be attributed to various factors, such as impaired leukocyte function with age and a reduced immune response [63]. Multiparous cows are repeatedly exposed to mastitis-causing pathogens, which increases the risk of developing subclinical mastitis [62]. Additionally, the teat canal diameter in multiparous cows may become more dilated with age due to previous injuries facilitating the entry of environmental and skin pathogens into the teat canal and, consequently, proliferation in the mammary tissue [64,65].

The distribution of the studied population showed that the Montbéliarde breed (*n* = 173) was highly present, from which 103 had subclinical mastitis (59.5%). Holstein cows were observed to be more susceptible to subclinical mastitis (68.2%), which aligns with previous studies [66,67,68]. However, crossbreed cows appear to be more resistant to mastitis infections than highly productive imported breeds. The high milk production capacity, intensive milking schedules and improper milking techniques contributed to the susceptibility and can further elevate the risk of mastitis in Holstein cows compared to more rustic crossbreed cows [38].

In the present study, 79.4% of CMT-positive cases were observed in cows at late lactation (≥7 months). Zaatout et al. [38] observed that the highest prevalence of subclinical mastitis (51.3%) was shown in the late stage and suggested that a long exposure to pathogens in the late stage of lactation increases the risk of subclinical mastitis. Otherwise, the type of pathogens involved in udder infection is influenced by the lactation phase, as previously suggested [55] and subclinical cases caused by *Streptococcus uberis* are more common than clinical cases, especially in late lactation, and are associated with higher somatic cell counts.

A high percentage (91.3%) of cows having subclinical mastitis produced less than 12 L/day of milk (low yield) compared to high-yielding cows (37.7%, *p* < 0.0001). In concordance with [38], the relationship between mastitis and the susceptibility of high-yield cows to develop mastitis remains unclear and requires further long-term investigations. However, previous studies [69,70] suggested that mastitis had an effect on decreasing milk production.

Regarding quarter-specific prevalence, we observed a higher incidence of mastitis in the rear quarters compared to the front quarters. These findings align with those of previous studies [71,72]. The reasons for the increased incidence of mastitis in the hindquarters remain unclear and not understood; some authors suggest that the anatomical position of the rear quarters, along with their greater exposure to urine and manure, may make them more prone to bacterial contamination [73,74]. In addition, cows with higher levels of dirtiness (scores of 2 or 3) were significantly more likely to test positive for subclinical mastitis compared to less dirty cows. A significant association between udder hygiene scores and leg hygiene scores (mean hygiene scores of 2 and 2.3, respectively), as well as a significant correlation between the prevalence of intramammary contagious pathogens and udder hygiene scores, has been reported [75].

Regarding the impact of milking method, no significant difference was observed between the incidences of subclinical mastitis in cows during mechanical milking compared to hand milking. It has been reported that machine milking was more frequently associated with subclinical mastitis [76,77]. However, these findings contrast with those of others, who reported a higher incidence of subclinical mastitis during hand milking compared to mechanical milking [66].

The use of mechanical milking is more practical in terms of time and labor efficiency, but it requires routine cleaning and disinfection of the machines. It is obvious that proper udder cleaning before milking is a critical preventive measure for mastitis and should be taken seriously by dairy cattle producers. In our study, a lack of proper Cleaning-In-Place systems for machine milking was observed on all farms. The absence of rigorous cleaning and post-milking teat dipping allows machine milking to easily transmit contagious pathogens from infected cows to healthy ones, which constitutes a key risk factor for subclinical mastitis. Furthermore, mechanical milking can impair the local defense mechanisms of the teat by altering its appearance and tissue, which increases susceptibility to intramammary infections [78]. In other contexts, hand milking is predominantly used on small and traditional farms. However, no significant differences were found between farm types (traditional vs. industrial) or stabling systems (tie stall vs. free stall) in terms of mastitis prevalence.

The current study showed that cows with subclinical mastitis experienced longer CFSIs and CCIs compared to healthy cows. In addition, cows with subclinical mastitis required more services per pregnancy and had a lower pregnancy rate at first service. Studies conducted in Argentina [79] and Chile [80] have shown poor reproductive performance in cows affected by subclinical mastitis. Mastitis can interfere with the resumption of ovarian activity and delay the first ovulation in postpartum dairy cows. It may also negatively impact reproductive performance by causing premature luteolysis or extending the follicular phase [19].

The results of the present study showed that cows with subclinical mastitis exhibited a significant increase in the number of NSPC and per pregnancy compared to healthy cows. This finding is consistent with studies, e.g., in Algeria, in Argentina and in Sri Lanka [1,36,79]. A decreased first-service pregnancy rate was observed in cows with subclinical mastitis; previous studies have also highlighted the link between mastitis and reduced pregnancy rates. Bouamra et al. [36] found that the first-service pregnancy rate was significantly lower in cows with subclinical mastitis. Lavon et al. [81] reported that approximately 30% of cows with subclinical mastitis experienced delayed ovulation. Direct alterations in ovarian function due to mastitis have been documented [82], with delayed ovulation associated with lower circulating estradiol concentrations and higher pre-ovulatory luteinizing hormone levels, both of which can negatively impact fertility [81].

In this study, the CFSI and CCI for subclinical mastitis-infected cows were found to be 28 and 40 days, respectively, longer than that of healthy cows. This result aligns with the findings of Bouamra et al. [36] in Algeria, where cows with subclinical mastitis experienced delayed first insemination compared to uninfected animals. Similar results were reported in Chile [80] and in Poland [83]. The negative impact of mastitis on reproductive performance, particularly the prolonged calving interval, may be linked to hormonal imbalances that disrupt follicular development. These imbalances can result in the release of substances that inhibit the expression of receptors for gonadotropins and other hormones necessary for reproduction activity [84].

Regarding the impact of pathogen type on the reproductive performance of cows, Gram-negative mastitis pathogens, particularly *E. coli* can induce a massive release of cytokines such as tumor necrosis factor-alpha (TNF-α), interleukin (IL)-1β, IL-8, and others [85,86]. The increase in inflammatory mediators is linked to reproductive failure during early lactation [87,88]. However, some pathogens may have a more significant impact on reproductive performance than others. This phenomenon of decreased reproduction was also induced by other pathogens. Both Gram-negative and Gram-positive pathogens may influence reproduction through similar mechanisms. For example, the peptidoglycan fragments of certain Gram-positive pathogens, such as staphylococci and streptococci species, can elicit immune responses similar to those caused by endotoxin infusion in coliform mastitis [87].

## 4. Materials and Methods

### 4.1. Description of the Study Area

The study was carried out in the Upper Cheliff plain. This region spans an area of approximately 375 km^2^, located 40 km from the Mediterranean Sea and 125 km southwest of Algiers, Algeria. It lies between longitudes 02°00′ and 02°25′ E and latitudes 36°10′ and 36°20′ N. The Dahra and Zeccar mountains border this region to the north, while the Ouarsenis mountain range forms its southern boundary [89]. This plain is characterized by a semi-arid Mediterranean climate and it receives about 430 mm of rainfall annually; the average temperature was estimated as 17 °C [90,91]. The total number of bovine livestock is about 27,407 heads in this region, mostly housed in mixed farms producing meat and milk, in which the number of dairy cows was estimated at 12,971 heads [92,93].

### 4.2. Study Design

The study was carried out in the region of the Upper Cheliff plain between December 2023 and May 2024. A cross-sectional design was chosen involving a total of 263 lactating cows housed in 23 farms. The farms whose owners provided verbal consent to participate in the study and agreed to sample collection were included. The farms were selected to represent all areas of the Upper Cheliff region and to ensure diversity in risk factors that could influence the occurrence of mastitis, such as legal status (private or state-owned), management system (modern or traditional), and milking method (mechanical or manual). Once a farm was included in the study, all lactating cows that showed no signs of clinical mastitis or other underlying diseases were enrolled and subjected to the CMT analysis.

The included cows in the present study were categorized into two classes: imported dairy cattle and improved dairy cattle, belonging to the breeds Holstein, Montbéliarde, Fleckvieh and cross-breed. Several data points, such as parity (primiparous, multiparous); the farming system (free-stall, mix-stall and tie-stall); the milking method (machine milking, hand milking); the daily milk production (<12 L; >12 L per day); the lactation stage (early, mid, late); animal cleanness scores; previous history of mastitis; and the application of disinfection before and after milking, were recorded. The cows’ diet primarily consists of a mixture of green and dry forages, supplemented with concentrated feed.

Before morning milking, the cow’s udders were carefully inspected and examined for the absence of the cardinal signs of udder inflammation or eventual changes in color, odor and consistency. Cows presenting symptoms of clinical mastitis were excluded from the study. Only cows with healthy udders were subjected to CMT in the post-colostrum period for screening subclinical mastitis. Each quarter that had no visible signs of clinical mastitis but showed a positive CMT result was considered to be affected by subclinical mastitis and subjected to milk sampling for microbiological analysis.

### 4.3. Mastitis Screening with California Mastitis Test

The CMT is a simple, economically feasible and efficient on-farm method used to screen subclinical mastitis in dairy cows. The CMT estimates the number of leukocytes in milk. After cell lysis by CMT reagent, the released DNA forms a gel, directly correlated with the numbers of somatic cells in the milk. The CMT reaction of each quarter was subjectively interpreted in an ordered scale as either 0, 1, 2, 3 or 4, with 0 signifying a negative reaction and 1 being a trace or a slight positive reaction; these scores were indicative of somatic cell levels (Table 7) [55].

After thoroughly cleaning the udders, a small amount of milk (about 3–5 mL) from each quarter was collected in the corresponding wells of a CMT paddle. Then an equal amount of CMT reagent (Raidex, GmbH, Dettingen an der Erms, Germany) was added to each well. The milk and reagent were gently mixed by swirling the paddle for a few seconds, the degree of gelling indicating the severity of mastitis. Results are graded on a scale from negative (no gel formation) to strong positive (severe gel formation) [94].

### 4.4. Collection of Milk Samples

Milk samples for microbiological analysis were collected from all quarters that scored positive on the CMT (score 1 or higher) (SCC ≥ 200,000 cells/mL). Briefly, the udders were carefully cleaned with water and dried with a disposable paper towel. The first few streams of milk were discarded to prevent potential contamination from inside the teat. The teats were then cleaned and disinfected using a tissue saturated with 70% alcohol (Avantor™ VWR Chemicals, Radnor, PA, USA). Approximately 20 mL of milk was collected from each quarter into separate sterile collection tubes. The tubes were labeled as front right (FR), front left (FL), rear right (RR) and rear left (RL). Detailed information about the cows and their farms, including cow number, stabling type, milking methods, age, lactation status, milk production and reproductive parameters, was recorded. Milk samples were promptly stored in an icebox at 4 °C and transported to the microbiology laboratory within 4 h.

### 4.5. Bacterial Isolation

A total of 317 quarter-milk samples aseptically collected from 155 lactating cows were subjected to bacteriological analysis. The isolation and preliminary identification of microorganisms were performed according to the National Mastitis Council [95] at the Microbiology Laboratory, Faculty of Life, Nature and Earth Sciences, University of Khemis-Miliana, Algeria. Briefly, a 10 µL drop of milk was streaked onto blood agar (Oxoid^®^, Basingstoke, UK) enriched with 5% sheep blood. For each sample, two plates were used; one plate was incubated aerobically and the second was incubated under anaerobic conditions at 37 °C for 24 to 48 h. The plates were then examined for macroscopic characteristics of colony morphology and hemolytic activity. Characteristic colonies (yellow or white; with hemolysis or without) were subsequently subcultured onto several selective media including, MacConkey Agar, Mannitol Salt Agar (Merck^®^, Darmstadt, Germany) and Baird Parker Agar (Oxoid^®^, UK). Pure cultures were subjected to preliminary identification using Gram staining and standard biochemical tests. Isolates were stored at −80 °C in tryptic soy broth (Merck^®^, Germany) supplemented with 30% glycerol until further use.

### 4.6. MALDI-TOF MS Identification

The identification of bacterial isolates obtained from milk samples was performed by matrix-assisted laser desorption/ionization time-of-flight mass spectrometry (MALDI-TOF MS) at the Friedrich Loeffler-Institute of Medical Microbiology, University Medicine Greifswald, Germany. Briefly, the frozen isolates were subcultured on BD BBL™ Columbia agar with 5% sheep blood (BD GmbH, Heidelberg, Germany) at 37 °C for 24 h. Sample preparation was carried out by the three different procedures recommended by the manufacturer: direct transfer (DT), extended direct transfer (eDT) and protein extraction (PE). Most of the samples were identified by the DT method or alternatively with eDT. For DT, a microbial biomass of a single pure colony was directly smeared on the spot of a MALDI target (MBT Biotarget 96, Bruker Daltonics, GmbH, Bremen, Germany) as a thin layer using a toothpick. Subsequently, the spots were overlaid by 1 µL of the α-cyano-4-hydroxycinnamic acid (HCCA) matrix (Sigma-Aldrich, Merck, Darmstadt, Germany). The eDT method was comparable to DT, but additionally 1 µL of 70% formic acid (Sigma-Aldrich, Merck, Darmstadt, Germany) was pipetted to the spots prior to matrix application. The PE method was performed for a few samples; for that, microbial biomass was transferred by an inoculation loop to a reaction tube containing 300 µL HPLC-grade water and vortexed. Then, 900 µL absolute ethanol (Sigma-Aldrich, Merck, Darmstadt, Germany) was added, and the tube was centrifuged for 2 min at 14,500 rpm (20,200× *g*). After discarding the supernatant, the tube was again centrifuged for 2 min at 14,500 rpm. The remaining supernatant was removed by pipetting, and the tubes were left open in air for 5 to 10 min. Next, 25 µL of 70% formic acid were added and the sample vortexed, followed by adding 25 µL of acetonitrile (Merck KGaA/Sigma-Aldrich, Darmstadt, Germany), vortexing, resuspending the pellet with a pipette, and again centrifuging for 2 min at 14,500 rpm. Then, 1 µL of supernatant was pipetted onto a spot of the MALDI-TOF MS target. Bacterial Test Standard (BTS, Bruker Daltonics, GmbH, Bremen, Germany) was used for each run for the instrument calibration. MALDI Biotyper^®^ sirius System with MBT Compass HT software, version 5.1.410 (Bruker Daltonics, GmbH, Bremen, Germany) has been employed for sample measurement and identification. The peak profiles of the isolates were compared to a reference spectra database with scores ranging from 0 to 3; a high-confidence identification was considered when a score of ≥2.0 was obtained. Isolates with scores lower than 2.0 (1.7–1.99) were classified as low-confidence identification. Such isolates were re-identified by the eDT method. If eDT did not deliver a score ≥ 2.0, we performed the PE method for such isolates. Isolates with a score < 1.7 were re-identified using the PE method as described previously [96,97].

### 4.7. Antimicrobial Susceptibly Testing

Antimicrobial susceptibility testing was carried out with the Vitek 2 system (bioMérieux, Marcy-l’Étoile, France) according to the manufacturer’s instructions. The Vitek cards AST-P654, AST-P655, AST-ST03 and AST-N428 were used for AST of staphylococci and mammaliicocci, enterococci, streptococci and *E. coli*, respectively. The interpretation of the results was performed according to CLSI breakpoints provided in guidelines [98]. The quality assurance and performance of antibiotic susceptibility testing were performed according to CLSI guidelines [98]. The reference strains *S. aureus* ATCC29213, *E. faecalis* ATCC29212, *S. pneumoniae* ATCC49619, *E. coli* ATCC25922 were used for quality control in this study. The disk diffusion method was used to perform antimicrobial susceptibility testing for both benzylpenicillin (P1) and cefoxitin (FOX) for the methicillin susceptibility test on *S. aureus* isolates, according to EUCAST guidelines.

The presence of *mec*A and *mec*C genes were detected by the Eazyplex^®^ MRSA test (Amplex Diagnostics, Gars-Bahnhof, Germany) on the Genie II device according to the manufacturer’s recommendations.

### 4.8. Data Processing and Statistical Analysis

Descriptive analysis and statistical tests were performed using IBM^®^ SPSS^®^ Statistics software, version 23 (IBM Corp., Armonk, NY, USA). A Chi-Square Goodness-of-Fit test was used to assess whether the frequency distributions of number, side position and CMT score of infected quarters significantly deviated from an equally expected distribution. To examine associations between potential risk factors (parity, cow breed, farming system, milking method, daily milk production, animal dirty score, lactation stage, previous SCM, disinfection before milking and disinfection after milking) and CMT results (positive vs. negative) Pearson’s Chi-squared test (χ^2^) was applied. Additionally, Student’s *t*-test was conducted to compare the means of reproductive performances between cows with and without SCM. Results were considered statistically significant at *p* < 0.05.

## 5. Conclusions

The present study, conducted in a dairy basin of Upper Cheliff in northern Algeria, revealed a prevalence of subclinical mastitis in 58.9% of cows and 31.1% at the quarter level. The study identified several risk factors contributing to subclinical mastitis, including parity, daily milk production, previous mastitis history, and animal cleanliness. Using MALDI-TOF MS, the most frequently recovered pathogens involved in subclinical mastitis were enterococci and CoNS, followed by *S. aureus* and *E. coli*. These findings underscore the importance of considering cows with subclinical mastitis as reservoirs for mastitis pathogens during monitoring and control efforts, as this can help track trends and inform targeted prevention measures.

The results further demonstrate that subclinical mastitis not only impacts milk production but also significantly affects the reproductive performance of dairy cows. The high prevalence of subclinical mastitis in this study is alarming and emphasizes the necessity to establish a national program for the early diagnosis and treatment of subclinical mastitis to prevent long-term repercussions on herd production and fertility. Additionally, improving hygiene and cleanliness in the animal environment, along with implementing strict milking protocols, is crucial to reduce environmental pathogens and minimize udder infections and mastitis.

Addressing subclinical mastitis from a One Health perspective is essential. This integrated approach not only strengthens veterinary public health efforts but also highlights the zoonotic potential of mastitis pathogens and the risk of antimicrobial resistance transmission between animals and humans.

Future investigations should incorporate SCC analysis to complement the CMT applied in the present study. The inclusion of SCC measurements will enhance diagnostic accuracy and provide a more comprehensive evaluation of subclinical mastitis. Furthermore, longitudinal study designs are required to elucidate the temporal dynamics and persistence of intramammary infections, extending beyond the cross-sectional framework employed here. Such approaches would also enable a more refined characterization of the microbiomes responsible for subclinical mastitis.

## Figures and Tables

**Table 1:** Prevalence of subclinical mastitis at quarters with CMT.

Variables		*n* (%)	Chi-2 Goodness	*p*-Value
Number of infected quarters per affected cow *n* = 155	One Tow Three Four	55 (35.5) 53 (34.2) 29 (18.7) 18 (11.6)	25.6	<0.0001
Side and position of infected quarter *n* = 317	Front Right Front Left Rear Right Rear Left	77 (24.3) 66 (20.8) 92 (29) 82 (25.9)	4.4	0.219
CMT Scores in infected quarters *n* = 317	1 (Traces) 2 (Weakly positive) 3 (Clearly positive) 4 (Strongly positive)	31 (9.8) 105 (33.1) 174 (54.9) 7 (2.2)	216.9	<0.0001

**Table 2:** Distribution of bacterial species isolated from CMT positive milk samples.

			CMT Score								Total
			1 Traces		2 Weakly Positive		3 Clearly Positive		4 Strongly Positive		
			Isolate 1	Isolate 2	Isolate 1	Isolate 2	Isolate 1	Isolate 2	Isolate 1	Isolate 2	
*S. aureus*		18	-	-	7	-	10	1	-	-	18
Non-*aureus* staphylococci and mammaliicocci	*Staphylococcus succinus*	2	-	-	2	-	-	-	-	-	45
	*Staphylococcus auricularis*	2	-	-	2	-	-	-	-	-	
	*Staphylococcus saprophyticus*	3	-	-	-	-	3	-	-	-	
	*Staphylococcus simulans*	8	-	-	2	-	6	-	-	-	
	*Staphylococcus xylosus*	2	1	-	-	-	1	-	-	-	
	*Staphylococcus hominis*	1	-	-	-	-	1	-	-	-	
	*Staphylococcus chromogenes*	5	-	-	3	-	2	-	-	-	
	*Staphylococcus cohnii*	1	-	-	1	-	-	-	-	-	
	*Staphylococcus haemolyticus*	8	1	-	3	1	2	1	-	-	
	*Staphylococcus felis*	1	-	-	1	-	-	-	-	-	
	*Staphylococcus epidermidis*	10	-	-	4	-	6	-	-	-	
	*Staphylococcus warneri*	1	-	-	1	-	-	-	-	-	
	*Mammaliicoccus sciuri*	1	-	-	-	-	1	-	-	-	
Enterococci	*Enterococcus durans*	1	-	-	-	-	1	-	-	-	125
	*E. faecalis*	52	6	-	11	2	23	7	2	1	
	*E. faecium*	62	6	6	20	5	15	8	1	1	
	*Enterococcus gallinarum*	2	-	-	-	-	1	1	-	-	
	*Enterococcus hirae*	7	-	-	-	2	4	1	-	-	
	*Enterococcus mundtii*	1	-	-	1	-	-	-	-	-	
Streptococci	*Streptococcus suis*	1	-	-	-	-	1	-	-	-	14
	*Streptococcus gallolyticus*	12	-	-	5	2	2	3	-	-	
	*Streptococcus infantarius*	1	-	-	-	-	-	-	1	-	
*Enterobacteriaceae*	*E. coli*	20	-	-	1	-	12	7	-	-	21
	*Citrobacter freundii*	1	-	-	-	1	-	-	-	-	
*Corynebacterium*	*Corynebacterium falsenii*	1	-	-	-	-	1	-	-	-	4
	*Corynebacterium flavescens*	1	-	-	-	-	1	-	-	-	
	*Corynebacterium provencense*	2	-	-	-	-	1	1	-	-	
*Bacillus*	*Bacillus mojavensis*	7	-	-	1	-	5	1	-	-	7
*Aerococcus viridans*		6	2	2	1	1	-	-	-	-	6
*Lactococcus*	*Lactococcus garvieae*	5	-	-	-	1	1	3	-	-	11
	*Lactococcus lactis*	6	-	-	1	-	3	2	-	-	
*Macrococcus caseolyticus*		1	-	-	-	1	-	-	-	-	1
*Bacillus* (*Niallia*) *circulans*		1	-	-	-	-	1	-	-	-	1
*Rothia* (*Micrococcus*, *Kocuria*) *kristinae*		1	-	-	1	-	-	-	-	-	1
Total isolates			16	8	68	16	104	36	4	2	254
Contaminated samples			-		6		12		-		18
Negative samples			11		21		38		2		72

**Table 3:** Antimicrobial susceptibility of bacterial isolates.

Antimicrobial Class	Antimicrobial Agent	Bacterial Isolates									
		*S. aureus* (18 isolates)		CoNS * (45 isolates)		Enterococci (125 isolates)		*E. coli* (20 isolates)		Streptococci (14 isolates)	
		R%	S%	R%	S%	R%	S%	R%	S%	R%	S%
Penicillins	Oxacillin	0	100	22.2	77.8	-	-	-	-	-	-
	Benzylpenicillin	0	100	-	-	-	-	35	65	0	100
	Ampicillin	-	-	-	-	0	100			0	92.3
	Piperacillin	-	-	-	-	-	-	40	60	-	-
Cephalosporins	Cefotaxime	-	-	-	-	-	-	0	100	0	92.3
	Ceftazidime	-	-	-	-	-	-	0	100		
	Cefuroxime	-	-	-	-	-	-	0	100		
	Ceftriaxone	-	-	-	-	-	-	-	-	0	92.3
Carbapenems	Ertapenem	-	-	-	-	-	-	0	100	-	-
	Imipenem	-	-	-	-	-	-	0	100	-	-
	Meropenem	-	-	-	-	-	-	0	100	-	-
Phosphonic acid	Fosfomycin	0	100	37.8	62.2	-	-	-	-	-	-
Potentiated sulfonamides	Sulfamethoxazole-trimethoprim	0	100	8.1	91.9	-	-	-	-	-	-
Glycopeptides	Vancomycin	0	100	0	100	0	100	-	-	0	100
	Teicoplanin	0	100	0	82.2	0	100	-	-	0	100
Fluoroquinolones	Levofloxacin	0	100	4.4	95.6	0.8	99.2	-	-	0	100
	Ciprofloxacin	-	-	-	-	-	-	15	85	-	-
Tetracyclines	Tetracycline	0	100	31.1	68.9	-	-	-	-	-	-
Lincosamides	Clindamycin	0	100	13.3	86.7	-	-	-	-	0	100
Aminoglycosides	Gentamicin	0	100	0	100	-	-	0	100	-	-
Rifamycins	Rifampicin	0	100	0	100	-	-	-	-	-	-
Macrolides	Erythromycin	0	100	15.5	84.5	-	-	-	-	0	100
Lipopeptides	Daptomycin	0	100	2.2	97.8	-	-	-	-		
Oxazolidinones	Linezolid	0	100	2.2	97.8	0.8	99.2	-	-	0	100
Nitrofuran	Nitrofurantoin	-	-	-	-	0	42	-	-	-	-
Streptogramins	Quinuiristin-dalfopristin	-	-	-	-	0.8	99.2	-	-	-	-

* including *Mammaliicoccus sciuri*, formerly *Staphylococcus sciuri*, a CoNS species.

**Table 4:** Association between risk factors and subclinical mastitis in tested cows.

Risk Factors	Level	N (%)	CMT Results		Chi-2 Dependency	OR (95%CI)	*p*-Value
			Positive N (%)	Negative N (%)			
Parity	Primiparous Multiparous	51 (19.4) 212 (80.6)	21 (41.2) 134 (63.2)	30 (58.8) 78 (36.8)	8.3	0.4 [0.2; 0.8]	0.004
Cow breed	Holstein Montbéliarde Fleckvieh Cross breed	44 (16.7) 173 (65.8) 13 (4.9) 33 (12.5)	30 (68.2) 103 (59.5) 6 (46.2) 16 (48.5)	14 (31.8) 70 (40.5) 7 (53.8) 17 (51.5)	4	-	0.267
Farming system	Free-stall Mix-stall Tie-stall	191 (72.6) 30 (11.4) 42 (16.0)	119 (62.3) 12 (40) 24 (57.1)	72 (37.7) 18 (60) 18 (42.9)	5.4	-	0.067
Milking method	Machine milking Hand milking	229 (87.1) 34 (12.9)	139 (60.7) 16 (47.1)	90 (39.3) 18 (52.9)	2.3	0.6 [0.3; 1.2]	0.131
Daily milk production	<12 L >12 L	104 (39.5) 159 (60.5)	95 (91.3) 60 (37.7)	9 (8.7) 99 (62.3)	74.7	17.4 [8.2; 37.1]	<0.0001
Animal cleanliness	Score-1 Score-2 Score-3	138 (52.5) 103 (39.2) 22 (8.4)	46 (33.3) 87 (84.5) 22 (100)	92 (66.7) 16 (15.5) 0 (0)	80.5	-	<0.0001
Lactation stage	Early Mid Late	106(40.3) 60 (22.8) 97 (36.9)	37 (34.9) 41 (68.3) 77 (79.4)	69 (65.1) 19 (31.7) 20 (20.6)	44.2	-	<0.0001
Previous history of SCM	Yes No	45 (17.1) 218 (82.9)	43 (95.6) 112 (51.4)	2 (4.4) 106 (48.6)	30.1	20.4 [4.8; 86.1]	<0.0001
Disinfection before milking	Yes No application	241 (91.6) 22 (8.4)	146 (60.6) 9 (40.9)	95 (39.4) 13 (59.1)	3.2	2.2 [0.1; 5.4]	0.073
Disinfection after milking	Yes No application	16 (6.1) 247 (93.9)	10 (62.5) 145 (58.7)	6 (37.5) 102 (41.3)	0.1	1.2 [0.4; 3.3]	0.765

**Table 5:** Impact of subclinical mastitis on fertility parameters in dairy cows.

Reproductive Parameters	Positive SCM	Negative SCM	*p*-Value
CFSI (days)	130.0 ± 39.9	102.7 ± 23.3	<0.0001
NSPC	2.5 ± 1.4	1.9 ± 1	
CCI (days)	164.7 ± 67.6	124.4 ± 28.3	
PRFS (%)	24.5	48.1	
≥3 services (%)	43.2	26.9	

SCM = subclinical mastitis; CFSI = calving-to-first-service interval; NSPC = number of services per conception; CCI: calving-to-conception interval; PRFS = pregnancy rate at first service; *p* = probability.

**Table 6:** Impact of the number of infected quarters on fertility parameters in dairy cows.

Reproductive Parameters	One-Quarter	Two-Quarters	Three-Quarters	Four-Quarters	*p*-Value
CFSI (days)	114.4 ± 27.2	127.9 ± 38.5	134.6 ± 35.5	176.8 ± 47.8	<0.0001
NSPC	1.9 ± 1.1	2.4 ± 1.1	2.8 ± 1.1	4.4 ± 1.9	
CCI (days)	135.8 ± 46.8	160.1 ± 61.4	175.7 ± 57.3	249.8 ± 82.3	

CFSI = calving-to-first-service interval; NSPC = number of services per conception; CCI: calving-to-conception interval; *p* = probability.

**Table 7:** Relationship of CMT scores to somatic cell counts [55].

CMT Score	Approximate SCC (cells/mL)	Gelling
0 (Negative)	0–200,000	None
1 (Traces)	200,000–400,000	Very mild
2	400,000–1 million	Mild
3	1–5 million	Moderate
4	>5 million	Heavy, almost solidifies

## Data Availability

All data supporting the findings of this study are presented within the paper.

## Abbreviations

AMR	Antimicrobial resistance
AST	Antimicrobial susceptibility testing
ATCC	American Type Culture Collection
BTS	Bacterial Test Standard
°C	Degree Celsius
CCI	Calving-to-Conception Interval
CFSI	Calving-to-First-Service Interval
CIP	Cleaning-In-Place
CLSI	Clinical & Laboratory Standards Institute
CMT	California Mastitis Test
CoNS	Coagulase-negative staphylococci
DT	Direct transfer
E	East
eDT	Extended direct transfer
EUCAST	European Committee on Antimicrobial Susceptibility Testing
FL	Front Left
FR	Front Right
HCCA	α-cyano-4-hydroxycinnamic acid matrix
HPLC	High-performance liquid chromatography
IL	Interleukin
L	Liter
MALDI-TOF MS	Matrix-Assisted Laser Desorption/Ionization Time-of-Flight Mass Spectrometry
*mec*A/*mec*C	Methicillin Resistance Genes
ml	Milliliter
MR-CoNS	Methicillin-Resistant Coagulase-negative staphylococci.
MRSA	Methicillin-Resistant *Staphylococcus aureus*
MSSA	Methicillin-Sensitive *Staphylococcus aureus*
N	North
NSPC	Number of Services per Conception
OR	Odds ratio
*p*	Probability
PE	Protein extraction
PRFS	Pregnancy rate at first service
RL	Rear Left
RR	Rear Right
SCC	Somatic Cell Count
SCM	Subclinical Mastitis
TNF-α	Tumor necrosis factor-alpha
χ^2^	Pearson’s Chi-squared test
